# Inward cholesterol gradient of the membrane system in *P. falciparum*-infected erythrocytes involves a dilution effect from parasite-produced lipids

**DOI:** 10.1242/bio.20147732

**Published:** 2014-05-29

**Authors:** Fuyuki Tokumasu, Georgeta Crivat, Hans Ackerman, Jeeseong Hwang, Thomas E. Wellems

**Affiliations:** 1 Malaria Genetics Section, Laboratory of Malaria and Vector Research, National Institute of Allergy and Infectious Diseases, National Institutes of Health, Bethesda, MD 20892-8132, USA; 2 Quantum Electronics and Photonics Division, Physical Measurement Laboratory, National Institute of Standards and Technology, Boulder, CO 80305, USA; * Present address: Department of Lipidomics, Faculty of Medicine, The University of Tokyo, 7-3-1 Hongo, Bunkyo-ku, Tokyo 113-0033, Japan

**Keywords:** Malaria, *Plasmodium falciparum*, Fluorescence lifetime imaging microscopy, Parasitophorous vacuole membrane, Maurer's cleft, Detergent-resistant membrane domain, Membrane rafts

## Abstract

*Plasmodium falciparum* (*Pf*) infection remodels the human erythrocyte with new membrane systems, including a modified host erythrocyte membrane (EM), a parasitophorous vacuole membrane (PVM), a tubulovesicular network (TVN), and Maurer's clefts (MC). Here we report on the relative cholesterol contents of these membranes in parasitized normal (HbAA) and hemoglobin S-containing (HbAS, HbAS) erythrocytes. Results from fluorescence lifetime imaging microscopy (FLIM) experiments with a cholesterol-sensitive fluorophore show that membrane cholesterol levels in parasitized erythrocytes (pRBC) decrease inwardly from the EM, to the MC/TVN, to the PVM, and finally to the parasite membrane (PM). Cholesterol depletion of pRBC by methyl-*β*-cyclodextrin treatment caused a collapse of this gradient. Lipid and cholesterol exchange data suggest that the cholesterol gradient involves a dilution effect from non-sterol lipids produced by the parasite. FLIM signals from the PVM or PM showed little or no difference between parasitized HbAA *vs* HbS-containing erythrocytes that differed in lipid content, suggesting that malaria parasites may regulate the cholesterol contents of the PVM and PM independently of levels in the host cell membrane. Cholesterol levels may affect raft structures and the membrane trafficking and sorting functions that support *Pf* survival in HbAA, HbAS and HbSS erythrocytes.

## INTRODUCTION

*Plasmodium falciparum* (*Pf*) malaria parasites remodel their host human erythrocytes to establish an environment suitable for their growth and replication. This remodeled environment includes the single bilayer membrane system of a parasitophorous vacuole (PVM) that surrounds the parasite as it develops and a tubulovesicular network (TVN) that extends from the PVM (Aikawa et al., 1986; Atkinson and Aikawa, 1990; Tilley et al., 2008). Ward et al. reported that the newly formed PVM connects with the host erythrocyte but some erythrocyte proteins are excluded from PVM (Ward et al., 1993). At the late schizont stage, the size of PVM approaches that of the host erythrocyte membrane (EM) (Diggs et al., 1977), while Maurer's clefts (MC) derive from the TVN in *Pf*-infected erythrocytes (Hanssen et al., 2008). Recent data support a role of MC for trafficking and sorting of exported parasite proteins en route to the EM (Spycher et al., 2006; Hanssen et al., 2008; Maier et al., 2009). Three dimensional (3D) electron tomography data indicate that a complex membrane system interconnects neighboring MC and may include connections to nearby vesicle-like structures (Tilley et al., 2008). A translocon complex termed PTEX recognizes export motifs (Marti et al., 2004) of many parasite proteins destined for host erythrocyte cytoplasm and membrane (de Koning-Ward et al., 2009); however, much remains unknown of the steps by which the PVM, TVN and MC develop and how shapes and biophysical properties of their membranes support trafficking of proteins and other molecules.

*Pf* parasites have no known de-novo cholesterol synthesis machinery (Sherman, 1979; Besteiro et al., 2010); nevertheless, cholesterol is vital to the properties of membranes including the domain assemblies and biological functions of lipid rafts (Lucero and Robbins, 2004; Mishra and Joshi, 2007; Lingwood and Simons, 2010). Rafts are involved in vesicular trafficking and signaling thought to be essential for *Pf* survival in erythrocytes (Marsh and Smith, 1973; Schreier-Muccillo et al., 1973; Presti, 1985; Samuel et al., 2001; Di Girolamo et al., 2008; Lingwood and Simons, 2010; Weber et al., 2010). Cholesterol concentration can influence membrane curvature (Chen and Rand, 1997) and may therefore affect the topology of parasite-derived membrane systems. Studies showing that cholesterol-rich domains are important for transfer of the major variable cytoadherence antigen (PfEMP1) to the host erythrocyte surface provide additional evidence for the role of cholesterol in protein trafficking and parasite survival (Frankland et al., 2006). However, details of raft dynamics during intracellular stages of *Pf* have not been fully understood. Direct observations of spatial and temporal distributions of cholesterol will provide important information on raft dynamics and their relationships to the parasite protein trafficking.

Protection of sickle-trait (hemoglobin S-containing) erythrocytes against malaria was reported by Allison almost 60 years ago (Allison, 1954). Although exact mechanisms of the protection are still not fully known, potential factors underlying the protective effect have been proposed, including higher cytoplasmic density from an altered condition of hydration, increased susceptibility to oxidant stress, and alterations of membrane lipid asymmetry (Kuypers, 2008). HbS in erythrocytes (Ham et al., 1968; Evans and Mohandas, 1987) may affect the ability of *Pf* parasites to introduce trafficking systems and remodel the host membrane with knob structures that enable pRBC to adhere in the microvasculature of brain and other organs (Cholera et al., 2008; Tokumasu et al., 2009; Cyrklaff et al., 2011; Kilian et al., 2013).

Here we report on membrane content variations between the host and parasite-installed membranes of *Pf*-infected HbAA, HbAS, and HbSS erythrocytes that can be observed directly by cholesterol detection methods including confocal, time-domain fluorescence lifetime imaging microscopy (FLIM) (van Munster and Gadella, 2005; Chang et al., 2007) with a fluorescent marker sensitive to cholesterol-rich domains. FLIM allowed direct, and high-resolution observations of relative cholesterol contents in each cell membrane compartment (change it to singular form) in live condition without disturbing membrane structures.

## RESULTS

### Florescence lifetime microscopy detects lipid differences between membranes

Upon invasion of an erythrocyte, the malaria parasite is surrounded by a PVM that incorporates elements of invaginated host membrane (Aikawa et al., 1981). The newly formed PVM is continuous with host erythrocyte but some erythrocyte proteins are excluded from PVM (Ward et al., 1993) by complex molecular events that occur at the membrane junction that forms during parasite invasion (Murphy et al., 2004; Cowman et al., 2012), suggesting that molecular organization of the PVM and parasitized EM (pEM) differ. To study the properties of membranes and cholesterol distributions in the pRBC and non-parasitized erythrocytes (nRBC), we used a membrane environment-sensitive fluorophore (1-[2-Hydroxy-3-(N,N-di-methyl-N-hydroxyethyl)ammoniopropyl]-4-[β-[2-(di-*n*-butylamino)-6-napthyl]vinyl]pyridinium dibromide (Di-4 ANEPPDHQ or “Di-4”)) (Obaid et al., 2004), which shows a blue-shift in emission spectrum of as much as 60 nm when it is inside a cholesterol-rich membrane phase (Jin et al., 2005). This membrane-binding fluorescent styryl dye is stably retained in the membrane (Obaid et al., 2004), enabling time-course observations of the fluorophore in the membrane for periods of several days without noticeable structural damage to the membrane. We also employed time-dependent fluorescence lifetime microscopy (FLIM) with time-correlated single photon counting (TCSPC) to detect the lifetime differences of Di-4 that are sensitive to local cholesterol content. For this purpose, the higher sensitivity and contrast of FLIM provided advantages over conventional spectral ratio imaging (Owen et al., 2006).

**Fig. 1.**
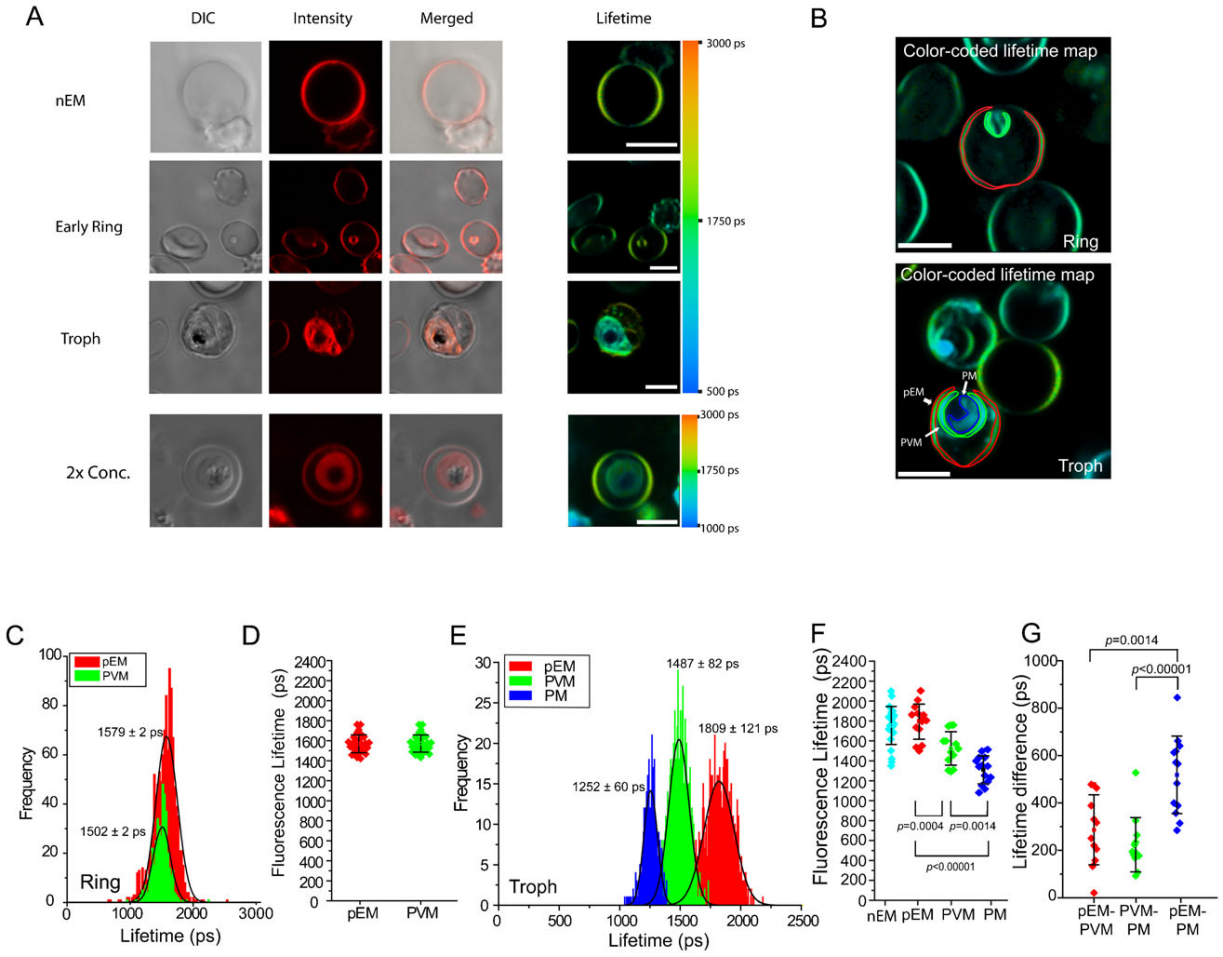
Fluorescence signals and fluorescence lifetime microscopy (FLIM) results from non-parasitized and pRBC. (A) Images and FLIM color maps of Di-4 labeled non-parasitized and pRBC. FLIM images are artificially colored from blue (500 ps) to orange (3000 ps) to display the differences and heterogeneities of fluorescence lifetimes in the membranes of the host erythrocyte, MC/TVN, PVM and parasite. (B) Regions from color lifetime maps of Di-4 ANEPPDHQ (Di-4)-labeled pRBC selected and exported for statistical analyses. PVM was carefully determined by overlaying DIC and lifetime maps. (C) Lifetime distributions from the pixel values from the membranes of a ring stage-infected erythrocyte. Each distribution was fitted with Gaussian curve to assess lifetime distributions. Fitting errors are less than 2 ps. (D) Distributions of average Di-4 lifetime values from the host membrane and PVM of ring stage-infected erythrocytes (3 independent experiments, total 29 cells analyzed). (E) Lifetime distributions from the pixel values from the membranes of a trophozoite stage-infected erythrocyte. (F) Distributions of average Di-4 lifetime values from the host and parasite membranes of trophozoite stage-infected erythrocytes. Lifetime distributions from the membranes of the host erythrocyte, PVM, and PM are statistically different (4 independent experiments, total 13 cells analyzed). (G) Distributions of differences between the average Di-4 lifetimes from membranes of the pRBC analyzed for panel F. DIC, differential interference contrast micrograph. Scale bars: 5 µm.

Fluorescence lifetimes from the pixels of imaged non-parasitized EM (nEM) were normally distributed about an average value of ≈1600–1800 ps (Fig. 1A). Exposure of the cells to a 2× higher concentration of Di-4 produced little or no change in these measured lifetimes (Fig. 1A), confirming that fluorescence lifetime was independent of fluorophore concentrations for these experiments. In our analyses, pixel lifetime values were recorded from the images and subjected to analysis as curves of fluorescence decay. Two component exponential decay functions were found to provide statistically better fits (chi-square) than single-component functions, consistent with the complexity of the host and parasite membranes relative to artificial membranes of simple lipid systems. The shorter lifetime contribution from two component analysis was typically below the 1850–3500 ps range found by Owen et al. in single component analysis of Di-4-labeled cholesterol-rich and cholesterol-free membranes of 1,2-dioleoyl-*sn*-glycero-3-phosphocholine and egg n-palmitoyl-sphingomyelin (Owen et al., 2006).

For quantitative analysis, Di-4 fluorescence lifetime values were obtained from pixels in selected regions of images of erythrocytes infected with parasites at different stages of their intraerythrocytic life cycle (Fig. 1B). For ring-infected erythrocytes, this analysis showed no significant lifetime difference between the pEM (1579±2 ps) and PVM (1502±2 ps) (peak ± fitting error) (Fig. 1C,D); however, for later stage trophozoite-infected erythrocytes, large and significant lifetime differences between the host, PVM and PM were consistently evident from cell to cell (pEM: 1820±191 ps, PVM: 1525±176 ps, PM: 1301±145 ps) (mean ± s.d.) (Fig. 1E,F). Differences between lifetime values in the pEM and the PVM (≈290 ps) were similar to the differences between lifetime values in the PVM and PM (≈230 ps); these two differences, added together, accounted for the large Di-4 lifetime difference between the erythrocyte and PM (≈500 ps) (*P*<0.00001) (Fig. 1G).

**Fig. 2.**
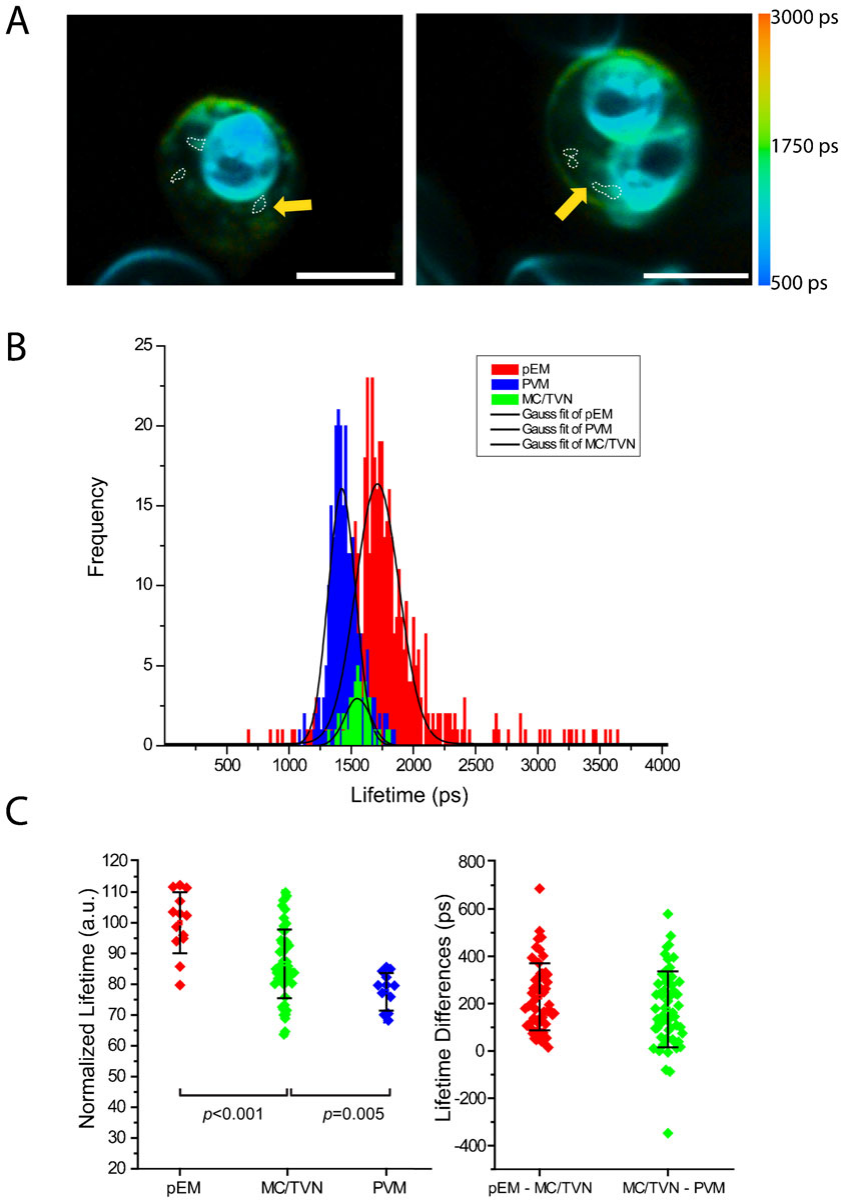
Fluorescence lifetime microscopy (FLIM) results from MC/TVN and PVM extensions (dotted lines) compared with the FLIM results from membranes of the host erythrocyte and PVM. (A) Color lifetime maps. (B) Histogram of lifetime distributions from host erythrocyte, PVM, and MC/TVN. (C) Distributions of average results and differences from the analysis of individual pRBC. Signals from MC/TVN were analyzed from a total 13 pRBC in 4 independent experiments. Scale bars: 5 µm.

Images of Di-4-labeled structures consistent with membranous structures induced in pRBC, such as MC/TVN could be distinguished in peripheral regions of the host erythrocyte (Fig. 2A). Analysis of the FLIM signals indicated that the lifetime values of the MC/TVN structures fell between those of the pEM and PVM (Fig. 2B). These lifetimes were on average 14% lower than those from the pEM but 10% higher than those from PVM (Fig. 2C, left, *P_pEM_*_-MC/TVN_<0.001, *P*_MC/TVN-PVM_ = 0.005, n_MC/TVN_ = 56), and the lifetime differences Δ_pEM-MC/TVN_ (229±142 ps, n = 56) and Δ_MC/TVN-PVM_ (176±160 ps, n = 56) (mean ± s.d.) were approximately the same (Fig. 2C, right). The intermediate values suggest a gradient in which MC/TVN cholesterol levels are lower than pEM cholesterol levels but higher than PVM cholesterol levels.

### Di-4 fluorescence lifetime values in pRBC are cholesterol-sensitive

**Fig. 3.**
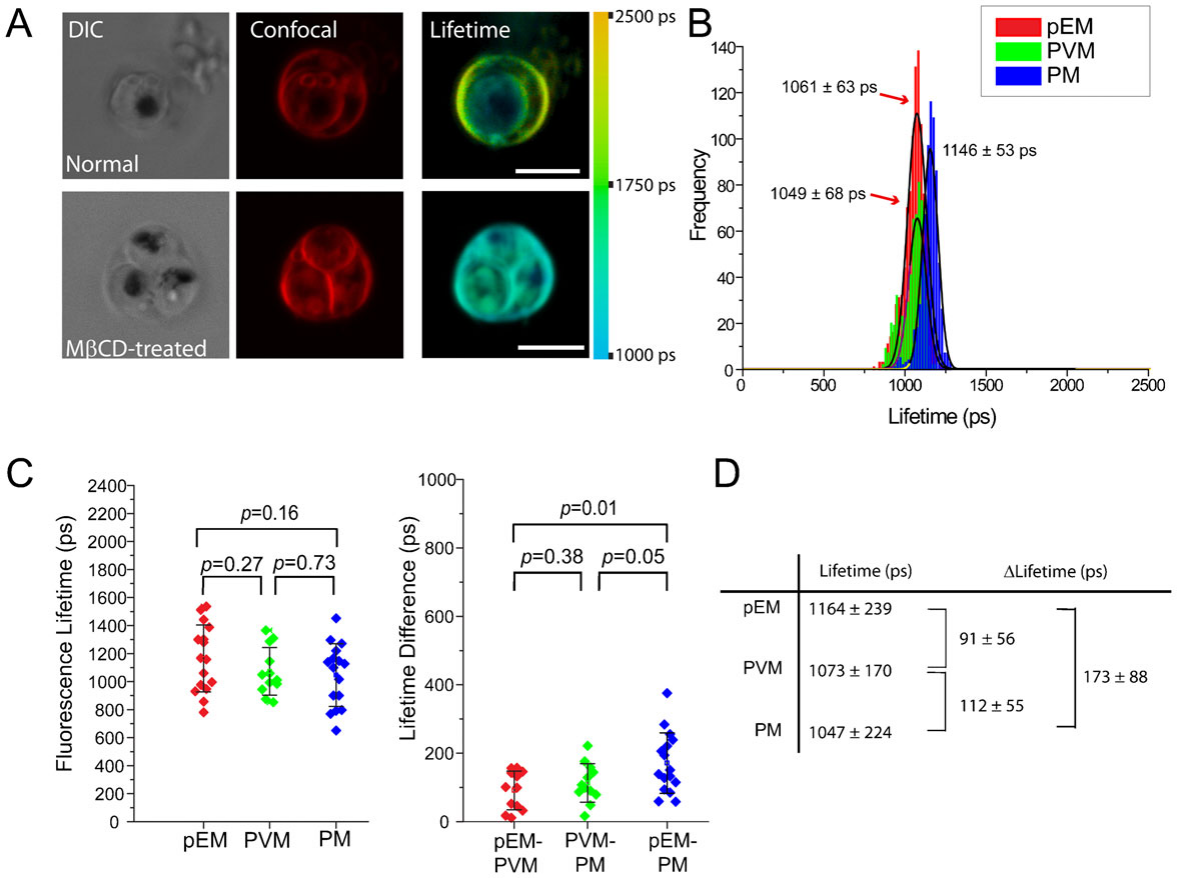
Effect of cholesterol removal on Di-4 ANEPPDHQ (Di-4) fluorescence lifetimes from the membranes of pRBC. (A) Images and color lifetime maps of untreated and methyl-β-cyclodextrin (MβCD)-treated pRBC. Cholesterol removal by MβCD causes a significant reduction of fluorescence lifetime that cannot be detected by regular confocal microscopy. We determined the MβCD concentration based on preliminary titration experiments. At this MβCD concentration, we did not observe any cell lysis due to the reduction of membrane cholesterol. (B) Histograms of the average lifetime values from an individual pRBC. (C) Distributions of the average lifetime values from the membranes of untreated and MβCD-treated pRBC (3 independent experiments, total 16 cells analyzed). Cholesterol removal greatly reduces the lifetime differences between the membranes of the host cell, PVM and parasite. (D) Table of Di-4 lifetimes and their differences from pRBC after treatment with MβCD (3 independent experiments, total 16 cells analyzed). Scale bars: 5 µm.

In a study of model membrane systems, Owen et al. reported Di-4 lifetimes of ≈3500 ps and ≈1850 ps, respectively, for cholesterol-rich and cholesterol-free membranes of 1,2-dioleoyl-*sn*-glycero-3-phosphocholine and egg *n*-palmitoyl-sphingomyelin (Owen et al., 2006). To confirm that Di-4 lifetimes reflect the cholesterol levels of membrane systems in pRBC, we depleted the cells of cholesterol by treatment with methyl-β-cyclodextrin (MβCD). Exposure of pRBC to 1.2 mM MβCD at room temperature for 30 min markedly changed their Di-4 lifetime profiles (Fig. 3A). These changes were evident in decreases of the average pEM fluorescence lifetime from 1820±191 ps (Fig. 1F) to 1164±239 ps (Fig. 3C, left), and of the PVM fluorescence lifetime from 1525±176 ps (Fig. 1F) to 1073±170 ps (mean ± s.d.) (Fig. 3C, left) (also compare Fig. 1E with Fig. 3B, histograms of lifetime distributions). MβCD treatment of the pRBC reduced the lifetime values of the pEM and PVM much more than the lifetime value of the PM, greatly collapsing the differences between the three membranes (Fig. 3C,D). These results offer further evidence that untreated cells have a higher cholesterol content in pEM than in the PVM, and that the PVM in turn has a higher cholesterol content than the PM.

**Fig. 4.**
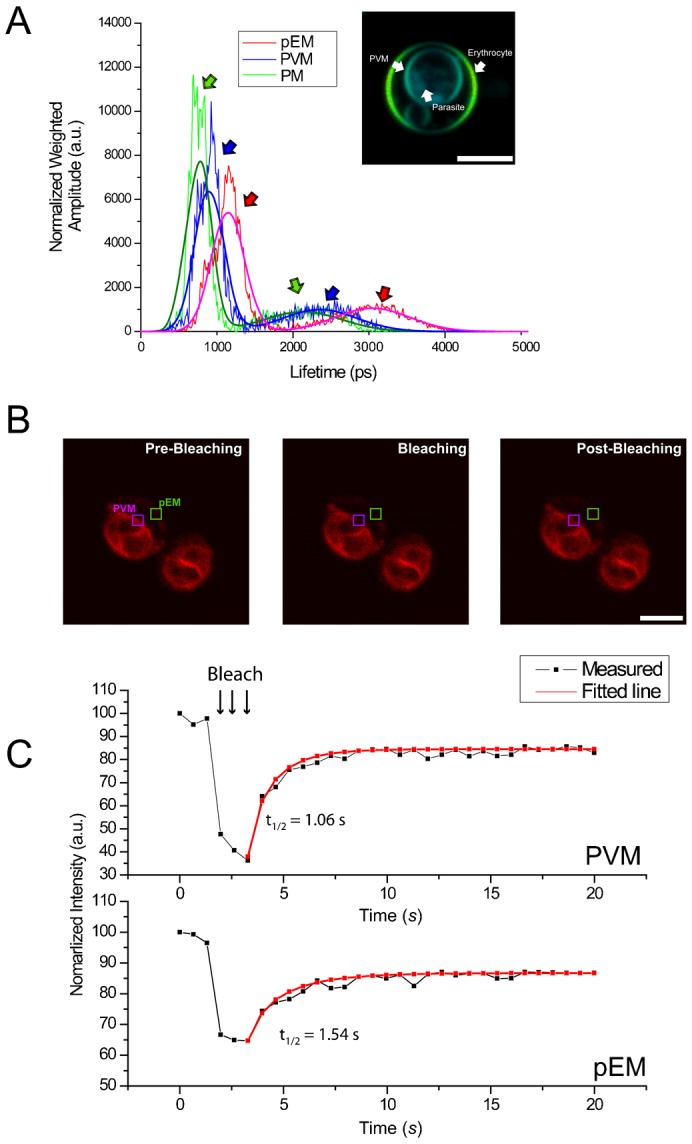
Fluorescence lifetime microscopy (FLIM) component analysis and fluorescence recovery after laser bleaching. (A) Each pixel value of fluorescence lifetime was decomposed into two components (fast and slow decay) with weighing values. Results of this mathematical decomposition are fit two Gaussian curves. (B,C) Bleached regions of pRBC (1.2 µm×1.2 µm). Images of the areas before, during and after bleaching are indicated by the boxes. (C) Fluorescence recovery curves. Three pre-bleaching, 3 bleaching and 25 post-bleaching frames were recorded (3 independent experiments, total 8 cells analyzed). Fluorescence recovered more rapidly from the parasitophorous vacuole membrane (PVM) than from the parasitized erythrocyte membrane (pEM). pEM, parasitized EM; PM, parasite membrane. Scale bars: 5 µm.

Biological membranes exhibit lateral heterogeneity in cholesterol content and include cholesterol-rich ordered phases (Owen et al., 2006). Fluorescence lifetime results from these membranes are collected as lifetime values from individual pixels in a multicomponent exponential decay function. For each pixel in two photon imaging, the FLIM value can be mathematically expressed as:
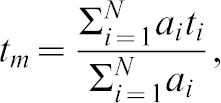
where *t_m_* is the average lifetime, *t_i_* (*t_1_*, *t_2_*, *t_3_* …) of single lifetime components, and *a_i_* (*a_1_*, *a_2_*, *a_3_*…) are relative amplitudes. Using multicomponent exponential decay fitting, we determined that lifetime pixel values for erythrocytes can be approximated with the weighted average of first two components (*t_i_ = t_1_*, and *t_2_*, *a_i_* = *a_1_*, and *a_2_*). To study how each component in this approximation contributes to the averaged lifetime of each pixel, we best-fit the lifetime pixel values to two components and plotted these in a histogram by their relative weights (*a_i_* in %) (Chia et al., 2008). For the pEM data, these components fell into two distributions: a relatively concentrated, high-amplitude distribution with a peak at 1144±1 ps and a second broad, low-amplitude distribution with a peak at 3044±17 ps (peak ± fitting error) (Fig. 4A). Corresponding peaks in the PVM data were present at 899±1.3 ps and 2354±19 ps; and peaks in the PM data were present at 758±0.95 ps and 2133±23 ps. The broad pEM distribution included a large population of long lifetime values >3000 ps in contrast to the shorter lifetime distributions from the PVM and PM (Fig. 4A). The full-width, half maximum (FWHM) estimates of concentrated, high-amplitude lifetime contributions were 270–430 ps, whereas those of broad low-amplitude (lower weight) contributions ranged from 1100–1200 ps. The peak heights of the high amplitude, short lifetime contributions also differed: for PVM and PM, these were approximately 10% and 25% less than from EM, respectively (Fig. 4A). Together, these differences of peak amplitudes suggest higher heterogeneity for the cholesterol-rich phases in the pEM than in the PVM or PM.

Changes of cholesterol content alter membrane phase behavior, fluidity, and protein diffusion (Orädd et al., 2002; Tokumasu et al., 2003; Marsh, 2009). To measure the fluidity of the membranes in pRBC, we employed fluorescence recovery after photobleaching (FRAP) on small areas of the pEM and PVM (Fig. 4B). After bleaching (to 30% of initial intensity) and removing the laser beam, fluorescence from PVM recovered to ≈70% of the original intensity with a *t*_½_ = 1.06 s, whereas fluorescence from EM reached a plateau more slowly (*t*_½_ = 1.54 s; Fig. 4C). These differences were statistically significant among multiple pRBC (PVM: 0.81 s *vs* RBC: 1.52 s, *P* = 0.029, n = 8) and are consistent with higher membrane fluidity and lower cholesterol content for the PVM than for the pEM.

### Malaria parasites may regulate cholesterol contents of the PVM and PM independently of different lipid content in the host membranes of HbAA and HbS-containing erythrocytes

**Fig. 5.**
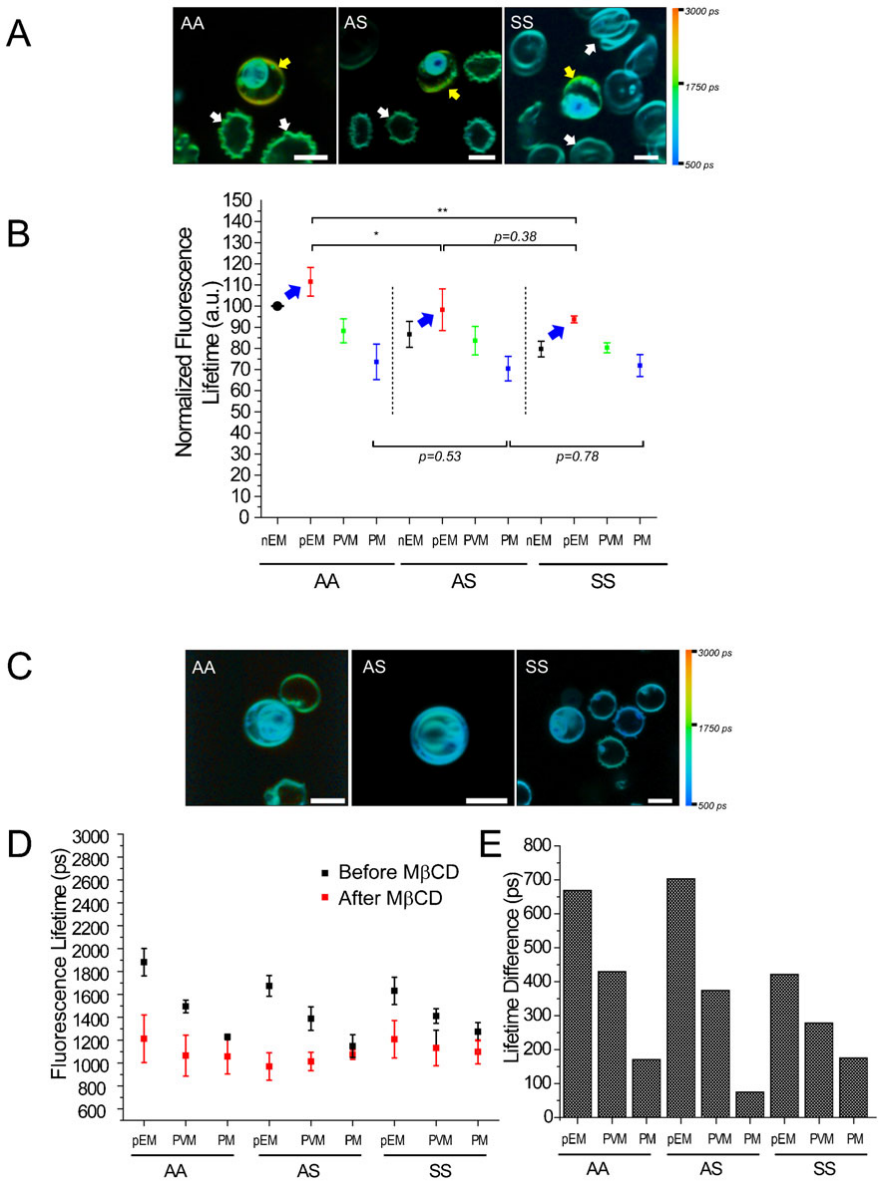
Di-4 ANEPPDHQ (Di-4) lifetime comparisons of non-parasitized and *P. falciparum* (*Pf*)-parasitized HbAA, HbAS, and HbSS erythrocytes. All comparisons were performed in parallel with fresh erythrocytes to exclude potential effects of blood aging or culture conditions. (A) Color lifetime maps of non-parasitized and *Pf*-parasitized HbAA, HbAS and HbSS erythrocytes. Yellow: pEM; white: nEM. (B) Distributions of lifetime values collected from experiments with four separate sample sets from different donors of HbAA, HbAS and HbSS erythrocytes. Eight cells of each blood type were analyzed in each experiment to obtain average values of lifetime. Each average lifetime value was normalized to the average lifetime from non-parasitized AA erythrocytes to compensate for experimental variation from culture conditions and microscope calibrations. (C) Color lifetime maps of methyl-β-cyclodextrin (MβCD)-treated non-parasitized and *Pf*-parasitized HbAA, HbAS, and HbSS erythrocytes. (D) Distributions of fluorescence lifetimes from the membranes of *Pf*-parasitized HbAA, HbAS and HbSS erythrocytes before (black) and after (red) MβCD treatment (n = 6 for each blood type). (E) Differences between the average Di-4 lifetimes from the membranes before and after MβCD treatment. Scale bars: 5 µm.

Differences in the membranes of HbS-containing and HbA-containing erythrocytes may affect important properties of *Pf*-infected cells (Hebbel, 1984; de Jong et al., 2001; Setty et al., 2002; Cholera et al., 2008). Comparative Di-4 fluorescence lifetime studies of non-parasitized and parasitized HbAA, HbAS, and HbSS erythrocytes offer one measure of these differences. Our fluorescence lifetime maps of Di-4 signals indicated comparatively shorter lifetime values from HbAS and HbSS erythrocytes than from HbAA erythrocytes (Fig. 5A). In further comparisons of non-parasitized HbAA, HbAS and HbSS erythrocytes from four independently collected samples under culture conditions (Fig. 5B), the Di-4 lifetime values from HbAS and HbSS erythrocyte membranes were 20% and 26% lower than from non-parasitized HbAA erythrocyte membranes (1674±91 ps (HbAS) or 1631±118 ps (HbSS) *vs* 1881±119 ps (HbAA). These results indicated comparatively lower cholesterol levels in the EMs of HbAS and HbSS relative to the EMs of HbAA erythrocytes (*P*<0.001: Bonferroni-Holm multiple comparison test; no statistical difference between the HbAS and HbSS EMs (*P_as-ss_* = 0.41)). We did not find any evidence for variations in overall fluorescence intensities from the different erythrocyte types, indicating that incorporations of the fluorophore were similar in all cells.

The EMs of pRBC consistently yielded longer Di-4 lifetimes than the EMs of nRBC in the same culture (Fig. 5B, blue arrows, two-way ANOVA, *P*<0.05), and significant lifetime differences were also evident between the EMs of parasitized HbAA, HbAS and HbSS erythrocytes (*P_aa-as_*<0.05 and *P_aa-ss_*<0.01). These data are consistent with relative increases of pEM cholesterol content after *Pf* parasitization of all three erythrocyte types. In contrast, no significant Di-4 lifetime differences were detected among the PVM or PM in parasitized HbAA, HbAS, and HbSS erythrocytes (*P*_pvm_aa-as_ = 0.24, *P*_pvm_aa-ss_ = 0.06, *P*_pm_aa-as_ = 0.52, *P*_pm_aa-ss_ = 0.71), suggesting that the PVM and PM cholesterol contents are regulated independently of host membrane cholesterol content.

Considering the evidence that Di-4 fluorescence lifetimes from HbAA erythrocytes are cholesterol-dependent (Fig. 3), we performed further experiments to compare the effects of cholesterol removal from HbAA, HbAS and HbSS erythrocytes. After MβCD treatment, pRBC of all types showed reduced average lifetime values of 1000–1200 ps from the pEM as well as the PVM and PM (Fig. 5C,D). We observed an increased number of crenated cells among the erythrocytes after MβCD treatment but no other morphological changes. Similar to results from MβCD-treated HbAA parasitized erythrocytes, MβCD treatment collapsed the lifetime differences between the host cell membrane, PVM and PM of parasitized HbAS and HbSS cells (Fig. 5D,E).

### Fluorescence signals transfer from the EM to parasite membranes following the invasion of Di-4 labeled erythrocytes by *P. falciparum*

**Fig. 6.**
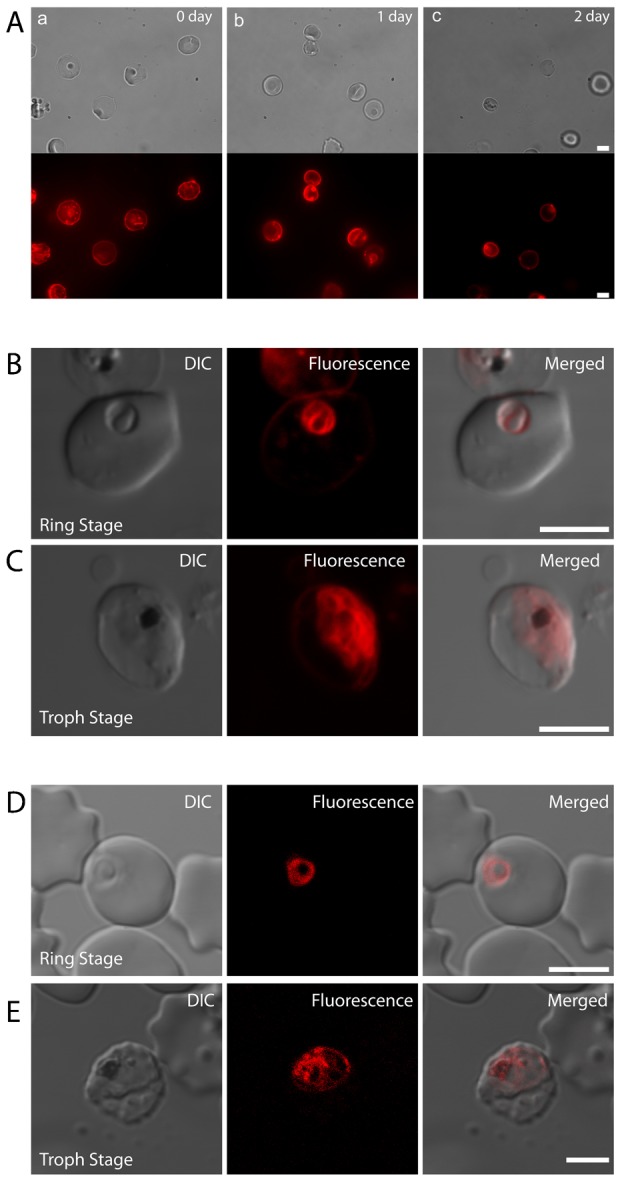
Lipid probe translocation from the host cell membrane to membranes of the parasitophorous vacuole and parasite in *Plasmodium falciparum*-infected erythrocytes. (A) Fluorophore stability test. Non-parasitized erythrocytes were labeled with Di-4 ANEPPDHQ (Di-4) and kept in culture condition for 48 hours. No structural damage by long term labeling with the fluorophore was observed from bright-field images. Epifluorescence images showed no apparent reduction in fluorescence intensity on both second and third day, verifying fluorophore stability in membrane. (B) Fluorophore translocation from labeled erythrocytes to non-labeled invading parasites. At 24 hours, the ring stage parasite and PVM are clearly labeled, while only weak fluorescence is present at the pEM. (C) Fluorophore signal from the PM and PVM indicative of continued probe translocation. (D) Fluorescence from an internal ring-stage parasite after invasion of non-labeled erythrocytes by merozoites released from labeled schizont-infected erythrocytes. No signal was observed from the host membrane of the ring stage-infected erythrocyte, suggesting that little or no translocation of the probe occurred from the PVM to the host EM. (E) Trophozoite stage parasite showing strong fluorescence from PM but little or no signal from the host erythrocyte. DIC, differential interference contrast micrograph. Scale bars: 5 µm.

To further study nature of the cholesterol gradient in pRBC, we probed for signals of Di-4 fluorescence that might provide evidence for transfer of cholesterol-rich complexes between the pEM and PVM. In one set of experiments, we combined Di-4-labeled nRBC with non-labeled, magnetically purified pRBC containing trophozoites and schizonts. This dye is stable in the nEM under culture conditions for at least 48 hours (Fig. 6A). After 24 hours, many Di-4-labeled erythrocytes were newly invaded with ring-stage parasites surrounded by bright fluorescence, indicating a flow of cholesterol-rich membrane components from the EM to the PVM during invasion (Fig. 6B). The pattern of fluorescence was indistinguishable from that of the newly formed PVM labeled by the lipophilic PKH26 probe and visualized by live video microscopy (Ward et al., 1993). Similar fluorescent patterns after probe internalization upon parasite invasion were also reported with DiI-C16 (Haldar and Uyetake, 1992).

The internalization of different membrane probes upon parasite invasion was previously found to be consistent with selective transfer of host erythrocyte lipids to the PVM (Dluzewski et al., 1995; Lauer et al., 2000; Lauer et al., 2001; Haldar et al., 2002; Murphy et al., 2007). Some fluorescence apparent inside the PVM (Fig. 6B) may also be from dyes that transfer from the PVM or pEM to the PM. Higher intensity of fluorescence from the PVM of the ring-stage parasite than from the host erythrocyte membrane may be a consequence of a concentration effect for some probes (Haldar and Uyetake, 1992). In our experiments with Di-4, the fluorescence patterns from trophozoite stages (imaged 24 hours after early ring-stages) revealed complex membrane structures within the PVM (Fig. 6C). These patterns are consistent with previous images of DiC-16 transfer from the pEM to developing parasites (Haldar and Uyetake, 1992).

Having observed Di-4 probe translocation from EM to the PVM after invasion, we wondered whether translocation in the opposite direction could also be demonstrated. Accordingly, in a second set of experiments, we combined magnetically-purified, late-stage Di-4-labeled pRBC with non-labeled, nRBC in culture. After allowing 1 d for the mature parasites to complete development and re-invade, we detected good fluorescence from newly-invaded ring stages no fluorescence from the surrounding host erythrocyte (Fig. 6D). These findings differ from those of Mikkelsen et al., who observed fluorescence from the surrounding host membrane as well as internal ring forms after merozoites from mature parasites labeled with 12-(9-anthroyloxy)oleic acid (12-AOle) were allowed to invade non-labeled erythrocytes (Mikkelsen et al., 1988). A possible explanation for this difference is that a detectable fraction of 12-AOle but not of Di-4 could move from the labeled parasite to the erythrocyte. Although the fluorescence patterns from the Di-4-labeled intraerythrocytic parasites became more complex as they matured, it remained the case that Di-4 fluorescence signal could not be detected from the membranes of host erythrocytes in our experiments (Fig. 6E).

### A fluorescent Bodipy-cholesterol analog also transfers to the PVM after parasite invasion of labeled erythrocytes

Major lipid classes of the EM include phosphatidylcholine, sphingomyelin, phosphatidylethanolamine, phosphatidylserine, phosphatidyl-inositol and cholesterol (Wang and Gustafson, 1994; Tokumasu et al., 2009). The absence of de novo cholesterol synthesis pathways from *Plasmodium* spp. requires growing parasites to incorporate cholesterol from the erythrocyte and blood environment (Sherman, 1979; Besteiro et al., 2010). We therefore employed Bodipy-cholesterol, a fluorescent cholesterol analogue with boron dipyrromethene difluoride linked to sterol carbon-24 (Li et al., 2006) to test for translocation of cholesterol from the pEM. Bodipy-cholesterol has been shown to behave similarly to native cholesterol in cultured vertebrate cells (Hölttä-Vuori et al., 2008) and to partition into liquid-ordered domains of model membranes enriched in cholesterol and sphingomyelin (Shaw et al., 2006; Li and Bittman, 2007). High photostability and quantum yield of its visible fluorescence spectrum make Bodipy-cholesterol a more robust probe for cholesterol trafficking studies than UV-excitable dehydroergosterol (DHE) (Hölttä-Vuori et al., 2008).

**Fig. 7.**
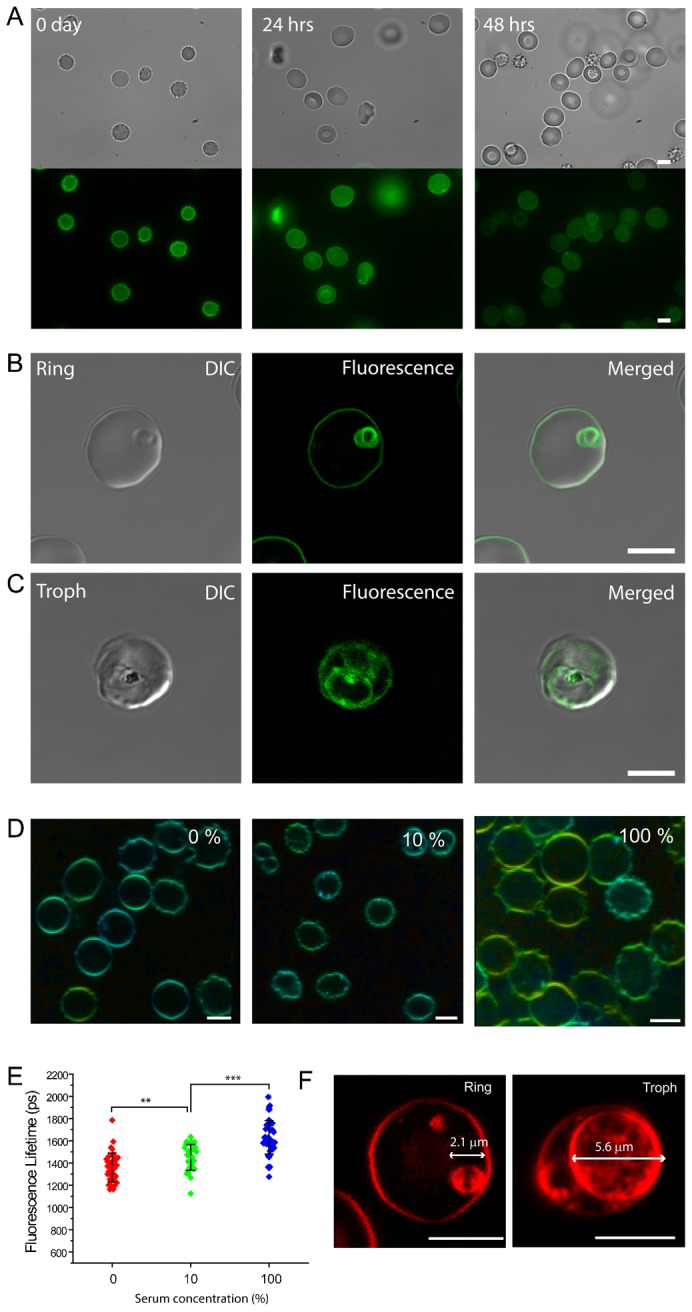
Boron-dipyrromethene (Bodipy)-cholesterol translocation from the EM to the PVM. (A) Stability test of Bodipy-cholesterol fluorescence from labeled, non-parasitized erythrocytes. No morphological changes were observed more than 48 hours after the addition of Bodipy-cholesterol. (B) Evidence for Bodipy-cholesterol transfer from the previously labeled erythrocyte to the membranes of invading non-labeled parasites. Unlike fluorescence from Di-4 ANEPPDHQ (Di-4)-labeled erythrocytes, Bodipy-cholesterol fluorescence from the host cell membrane remains readily detectable after parasitization. (C) Trophozoite stage-infected erythrocytes show Bodipy-cholesterol fluorescence from the PVM and its TVN extensions in the erythrocyte cytoplasm, but fluorescence is no longer evident at the pEM. Note also the absence of fluorescence from the interior of parasite itself. DIC, differential interference contrast micrograph. (D) Di-4 FLIM signals from non-parasitized HbAA erythrocytes incubated in 0, 10, 100% human serum for 48 hours after MβCD treatment. (E) Di-4 FLIM lifetime value distributions after each 48 hours incubation; lifetime recovery depends upon serum concentration. The number of RBC analyzed for 0, 10, and 100% serum were 38, 33, 48, respectively. (F) Images showing PVM sizes of ring (left) and trophozoite (right) stages in pRBC. Scale bars: 5 µm.

Methods employing either methyl-*β*-cyclodextrin/Bodipy-cholesterol complexes or a simple mixture of Bodipy-cholesterol in erythrocyte suspensions were found to provide effective labeling, and the fluorescence from labeled erythrocytes was stable for more than 24 hours under culture conditions (Fig. 7A). Slight decreases (≈20%) of fluorescence intensity were detected after 48 hours, but this intensity remained adequate for confocal microscopy. We evaluated fluorescence from newly-invaded erythrocytes in samples prepared by combining Bodipy-cholesterol-labeled erythrocytes with non-labeled schizont-parasitized erythrocytes. As with the Di-4 of labeled erythrocytes invaded by non-labeled parasites (Fig. 6B), Bodipy-cholesterol transferred internally to the parasite membranes, although this transfer was less rapid: fluorescence from the pEM of the Bodipy-cholesterol-labeled erythrocytes persisted for up to 24 hours after invasion (Fig. 7B). With further progression to fully-developed trophozoite stages (≈36 h after invasion), the Bodipy-cholesterol fluorescence decreased from the pEM, while fluorescence continued to spread with the growing PVM and exo-PVM membrane system inside the erythrocyte (Fig. 7C). These provide further evidence for transfer of cholesterol from the pEM to the PVM and the PM after invasion. We observed no morphological abnormalities of schizonts in the presence of Bodipy-cholesterol dye, but exit of merozoites from mature schizonts did not occur, perhaps because of an effect of Bodipy-cholesterol in the ordered process of egress (Blackman, 2008).

Surrounding serum may supply cholesterol to the pEM (Grellier et al., 1991). To confirm this phenomenon by the methods of our study, we incubated MβCD-treated nRBC in human serum (natural source of cholesterol) for 48 hours at 37°C (Fig. 7D). A statistically significant, partial recovery of the Di-4 lifetime was observed after incubation in 10% serum, and full recovery up to the normal range of ≈1800 ps was observed in 100% serum (Fig. 7E).

We also performed mathematical estimations from PVM to better understand how FLIM signal might be influenced by parasite growth. The early ring-stage PVM (Fig. 7F, left) has a diameter of ≈2.1 µm and a calculated surface area of 13.8 µm^2^ when a spherical model is applied. When PVM expands to a diameter of 5.6 µm at trophozoite stage (Fig. 7F, right), the surface area increases to approximately 97.4 µm^2^, ≈7.1 times more than that of the ring stage PVM. This increase in surface area is accompanied by the addition of non-sterol lipids from parasite that significantly reduces the relative cholesterol level of the PVM.

## DISCUSSION

Growth of the intraerythrocytic malaria parasite is accompanied by an intense period of membrane biogenesis including production of a vacuolar system that surrounds and supports the parasite's expansion in the host cell (Vial et al., 1990). The processes of membrane engineering that underlie this biogenesis begin with parasite invasion of the erythrocyte and continue with development of the surrounding PVM, TVN extensions into the host cell cytoplasm, MC and small vesicles that may move between some of these structures and the host membrane (Aikawa, 1988; Taraschi et al., 2003; Bhattacharjee et al., 2008; Hanssen et al., 2008; Kilian et al., 2013).

The human erythrocyte, although a naturally non-endocytic cell, is induced by the malaria merozoite to invaginate for incorporation of the young parasite into the sealed PVM (Miller et al., 1979). A number of studies have found that the newly formed PVM includes host membrane lipids that flow past the erythrocyte–merozoite moving junction and leave many of the host intramembranous particles and proteins behind (Langreth, 1977; Aikawa et al., 1978; Miller et al., 1979; Aikawa et al., 1981; Mitchell and Bannister, 1988; Ward et al., 1993). Lipid additions from merozoite rhoptries to the new invaginated vacuolar membrane have also been proposed (McLaren et al., 1979; Stewart et al., 1986; Mikkelsen et al., 1988); however, the lipid mass of these additional contributions is thought to be relatively small in light of evidence from surface area measurements that the early PVM of *Toxoplasma* is primarily from invaginated host membrane and contains only 0–18.5% parasite-derived material (Suss-Toby et al., 1996). This predominance of host–erythrocyte over parasite-contributed lipid in the invaginated vacuolar membrane of the early ring is supported by two findings from our study. First is the evidence for fluorescent lipid transfer from labeled erythrocytes to the membranes of parasites upon invasion. Second – and perhaps more strong – is the evidence that cholesterol levels in the newly formed PVM of early ring stages are similar to those of the pEM. Only later in the intraerythrocytic parasite life cycle does the relative cholesterol content decrease in the PVM of the fully internalized vacuole as it expands to envelop the larger growing parasite.

FLIM data show stepwise reductions of Di-4 fluorescence lifetimes from the host pEM to MC/TVN, MC/TVN to the PVM, and from PVM to PM, suggesting successive decreases of the cholesterol contents in these membranes. These findings are consistent with: (1) early PVM origination from the host EM; (2) lack of cholesterol synthesis by the intra-erythrocytic malaria parasite; (3) intense incorporation into the parasite membranes of parasite-synthesized phospholipids (especially phosphatidylcholine and phosphatidylethanolamine), most of which are produced from host-supplied precursors (Holz, 1977; Sherman, 1979; Grellier et al., 1991; Hsiao et al., 1991; Palacpac et al., 2004; Vial et al., 2005; Tarun et al., 2009; Besteiro et al., 2010); and (4) uptake and incorporation of cholesterol from the pEM into the membrane systems installed by the growing parasite within the erythrocyte. Variations of cholesterol level among the membranes of pRBC have also been suggested by spectral shifts in Nile Red as a neutral lipid indicator (Jackson et al., 2004). Recently, parasite proteins which can transfer various phospholipids have been identified, suggesting potential participants for the molecular regulation of lipid levels (van Ooij et al., 2013).

Membrane fluidity is affected by cholesterol content as well as lipid and protein composition (Alberts et al., 2002). Our observations of faster recoveries of fluorescence in PVM after local photobleaching are consistent with higher fluidity and more rapid lateral probe movement in membranes with lower cholesterol levels. Other factors that may affect membrane fluidity involve changes in relative concentrations of sphingolipids, phospholipid with unsaturated acyl chains, and mixture ratio of lipids with low and high melting (phase transition) temperatures. Lipid species distributions may relate to the bending modulus of the parasite membranes including the PVM, TVN and MC, as lipids with larger or smaller head groups have been shown to prefer more or less pronounced membrane curvatures, respectively (Song and Waugh, 1993; Pan et al., 2009). Several protein families and intramembrane protein interactions are known to be involved in the structure and curvature of the ER in eukaryotic cells (Hu et al., 2009; Park and Blackstone, 2010). While they have yet to be characterized in malaria parasites, we hypothesize that such proteins may contribute to the architecture of the PVM, TVN and MC and that they may be important determinants of cholesterol and lipid distributions in pRBC.

Cholesterol-rich raft regions of the pEM, TVN, MC and PVM may influence protein function and targeting (Murphy et al., 2006). While major proteins of EM rafts such as stomatin and band 3 appear to be excluded from the growing parasite vacuole, flotillin-1, flotillin-2 and at least eight other erythrocyte raft proteins are recruited to the PVM (Murphy et al., 2004). This discriminating internalization of raft proteins, the relative paucity of intramembranous particles observable in PVM by freeze fracture electron microscopy (McLaren et al., 1979; Aikawa et al., 1981), and the low cholesterol levels of both PVM and PM invite fundamental questions about the role of raft proteins in the pRBC. Cholesterol's association with proteins in rafts contributes to their detergent extraction resistance and provides the basis for the isolation of regions of detergent-resistant membrane (DRM) (Simons and Ikonen, 1997; Brown and London, 1998); despite the overall lower relative cholesterol level of the PVM, raft DRM of the PVM occur in association with parasite-produced proteins as well as proteins recruited from host membrane (Lauer et al., 2000; Yam et al., 2013). Rafts may function in the sorting of lipids and proteins in vacuolar and secretory pathways, and they may have roles in the structure and properties of the internal PMs that affect such processes as endovacuolation, host cell hemoglobin consumption, and macromolecular transport (Haldar et al., 2001). Indeed, reductions of cellular cholesterol or sphingolipid contents have been shown to cause mis-sorting of GPI-anchored proteins, of an influenza protein and of other membrane proteins (Mayor and Maxfield, 1995; Keller and Simons, 1998; Ledesma et al., 1998; Mayor et al., 1998; Nyasae et al., 2003; Procino et al., 2010). In addition to their participation in lipid and protein trafficking of the *Pf*-infected erythrocyte, cholesterol-rich rafts at the host cell surface may have roles in parasite invasion, signaling pathways and cytoskeletal reorganization events (Samuel et al., 2001; Harrison et al., 2003; Murphy et al., 2004).

**Fig. 8.**
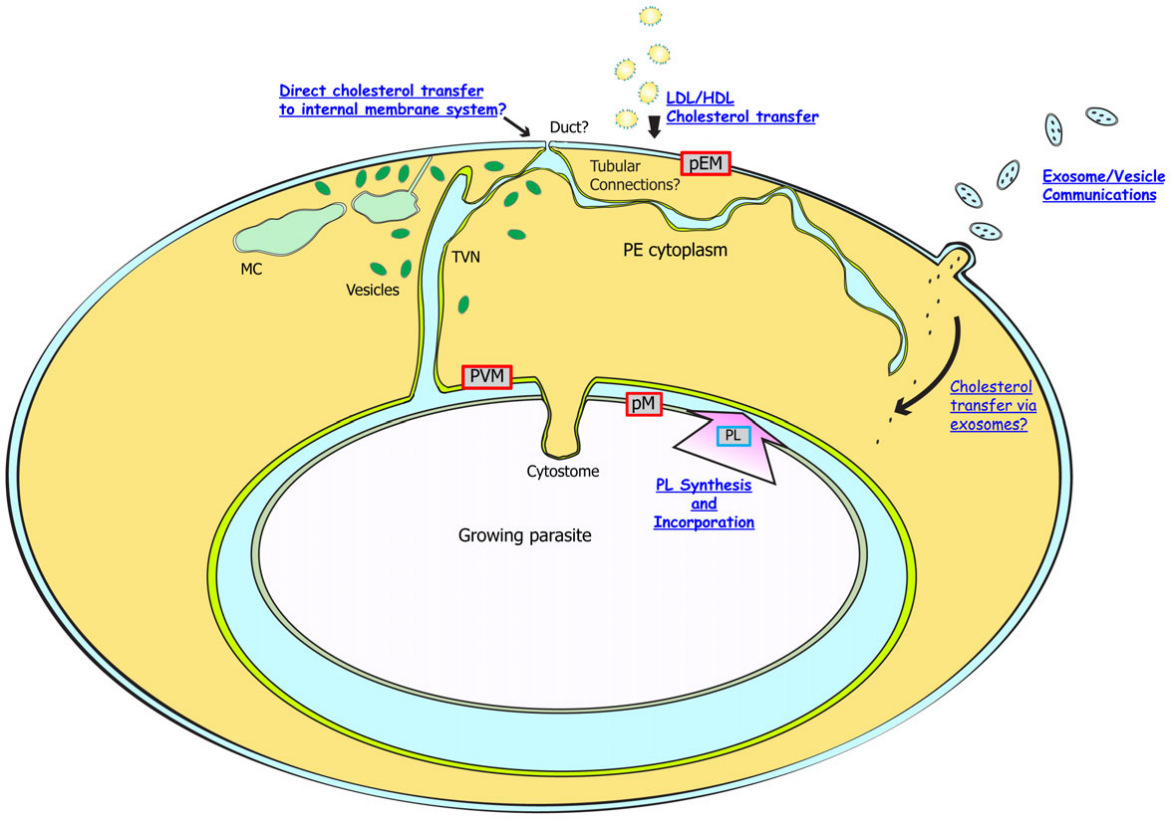
Model for cholesterol gradient development in the pRBC. The initial, early ring-stage PVM formed during parasite invasion has the same cholesterol level as the pEM. The growing parasite synthesizes and supplies large amounts of phospholipids to the PVM, accommodating the size increase of the PVM and diluting its cholesterol concentration. Cytostome formation to internalize host hemoglobin may deliver PVM cholesterol into parasite. Some cholesterol may also be transferred from the pEM to the PVM through the physical contacts between pEM and intracellular membrane system including MC and the TVN. Vesicles associated with the TVN and MC may contribute to cholesterol delivery. Serum cholesterol may also transfer to membrane systems of the intraerythrocytic parasite by exosomes involved in cell-to-cell communication or by direct physical connections with the host cell membrane.

Our observations suggest that cholesterol may transfer from the pEM to the PVM, as: (1) Bodipy-cholesterol as well as Di-4-cholesterol complexes can transfer from the EM to internal membranes of *Pf*-infected cell as the intraerythrocytic parasite matures; and (2) by FLIM analysis, the cholesterol level of the PVM is midway between levels in the pEM and PM, suggestive of an internal gradient from an ongoing uptake process. The evidence for expanded fluorescence patterns and brightly labeled PVM in Di-4- and Bodipy-cholesterol-labeled erythrocytes infected by mature-stage parasites is consistent with the uptake of cholesterol from the pEM. In contrast, the lack of fluorescence from the host membranes of non-labeled erythrocytes after infection by Di-4-labeled parasites suggests comparatively poor transfer of cholesterol from the PM to host pEM, as is schematically depicted in Fig. 8. Fluorescence signals from the internal parasite membrane system at trophozoite stage suggest a transfer mechanism that internalizes the PVM components into the parasite. The double-membrane cytostome (Aikawa et al., 1966) could contribute to this transfer by its action to deliver hemoglobin from the pRBC cytoplasm to the parasite digestive vacuole.

Interestingly, membrane structures that originate at the periphery of TVN (Hanssen et al., 2008) showed FLIM lifetimes that are intermediate between pEM and PVM. These differences between MC/TVN and PVM, and evidence from reconstructed electron microscopy data showing the possibility of physical connection between pEM and MCs (Hanssen et al., 2010), offer support for a model whereby some cholesterol is recruited from the erythrocyte membrane to MC/TVN. Possible contributors to cholesterol transfer include direct cholesterol incorporation from surrounding serum through the TVN and proposed ‘duct’ opening (Pouvelle et al., 1991), lipoprotein attachment (Grellier et al., 1991), vesicle-like structures that are associated with MC and communications between pEM and MC (Trelka et al., 2000; Kriek et al., 2003; Hanssen et al., 2008), and erythrocyte-derived microvesicles that have been implicated in cell–cell communications between pRBC (Mantel et al., 2013; Regev-Rudzki et al., 2013).

Our FLIM data indicate that the membranes of HbAS and HbSS erythrocytes in this study were lower in cholesterol than the membranes of HbAA erythrocytes. Despite these differences, similar cholesterol levels were observed in the PM and in the PVM of the HbS-containing and HbAA erythrocytes infected by trophozoites. These findings suggest a mechanism of regulation that can maintain the cholesterol levels of *P. falciparum* membranes in host cells of different lipid composition and are consistent with the importance of these levels for membrane maintenance and function in the pRBC.

## MATERIALS AND METHODS

### Cell culture

Adult subjects provided written informed consent in accordance with the Declaration of Helsinki and were enrolled at the National Institutes of Health Clinical Center on clinical protocol NIH 03-H-0015 specifically approved for this study by the Institutional Review Board of the National Heart, Lung and Blood Institute. Erythrocytes from HbAA, HbAS and HbSS volunteers at the National Institutes of Health Clinical Center were drawn into Vacutainers® containing acid-citrate-dextrose anticoagulant. After removing buffy coat leukocytes, erythrocytes were washed three times with Roswell Park Memorial Institute (RPMI) 1640 (Invitrogen, Carlsbad, CA) and stored at 50% hematocrit at 4°C prior to use (within 4–36 h of blood draw). Alternatively, for experiments employing only HbAA erythrocytes, O+ erythrocytes were purchased from the Interstate Blood Bank, passed through a Sepacell R-500 filter (Baxter, Deerfield, IL) to remove leukocytes and platelets, washed, re-suspended in RPMI 1640 medium (Invitrogen), and stored at 4°C.

*Pf* (3D7) parasites were cultivated in human erythrocytes (5% hematocrit) in RPMI 1640 medium supplemented with 0.5% (w/v) Albumax II (Invitrogen), 2 mg/ml sodium bicarbonate (Invitrogen), 0.10 mM hypoxanthine (Sigma–Aldrich, St Louis, MO), 25 mM Hepes and 10 mg/ml gentamicin (Gibco, Carlsbad, CA) at 37°C. To establish similar parasitemia between HbAA, HbAS, and HbSS cells for comparative purposes, double the number of purified parasites was added to HbSS erythrocytes than to the HbAA or HbAS erythrocytes.

### Fluorescence labeling

pRBC containing late stage parasites were magnetically isolated using MACS® separation columns (Miltenyi Biotec, Auburn, CA), washed and resuspended in 20 mM HEPES buffered saline (HBS), pH 7.05. For removal of pEM cholesterol, 3.5 mM of MβCD (Sigma–Aldrich) in HBS was added to the medium. Di-4 ANEPPDHQ (Invitrogen) in dimethyl sulfoxide (DMSO) was added to a concentration of 3 µg/ml, and erythrocytes at ≈0.1 hematocrit were incubated for 30 min at RT. For cholesterol labeling, 5 µl of 1:1 molar ratio (2 mM:2 mM) of MβCD:Bodipy-cholesterol (Avanti Polar Lipids, Alabaster, AL) was mixed with 45 µl of incomplete RPMI and incubated with 5 µl of erythrocytes at 50% hematocrit at 37°C for 2 h. Alternatively, erythrocytes were labeled with 2 µM of Bodipy-cholesterol in incomplete RPMI (without MβCD) for 40 min at 37°C. Erythrocytes were washed with incomplete RPMI three times to remove excess probe. For membrane label exchange studies, labeled or non-labeled erythrocytes were incubated either 24 or 48 h with non-labeled pRBC or labeled matured parasites, respectively.

### Fluorescence microscopy and FLIM

Cells were washed with HBS three times to remove the phenol red of RPMI medium before fluorescence microscopy. FLIM data were collected using a Becker and Hickl time-correlated single photon counting SPC 830 fluorescence lifetime system connected to a Leica SP5 confocal microscope (Leica Microsystems, Bannockburn, IL). Two-photon excitation of fluorescence was performed with a Maitai^TM^HP Ti:Sapphire Laser (Spectra-Physics, Newport, Santa Clara, CA) tuned to 910 nm with 100 fs pulses at a rate of 80 MHz. The optimal two-photon excitation wavelength (910 nm) was determined by separate experiments testing a wide range of wavelengths. Images were acquired using a 63× NA 1.4 objective lens. Quantitative analysis of lifetime data was by SPCImage software (v2.9.9) from Becker and Hickl. Epifluorescence data were collected with Leica DMI6000 (Leica Microsystems) using 100× NA 1.4 objective. Pixel-based fluorescence lifetime data were exported in text image format for statistical analyses of lifetime values by Origin 8 Pro SR6 software (Origin Lab, Northampton, MA), Image J, and MATLAB (The MathWorks, Inc., Northampton, MA). Confocal Image data were analyzed by Image Pro 6.3 software (Media Cybernetics, Bethesda, MD). Post-FRAP data were collected with Leica SP2 confocal microscope (Leica Microsystems) at a scan rate of 1000 Hz per line using 488 laser line for excitation and emission window at 550 nm–600 nm combined with beam expander 3.

### Abbreviations

12-AOle, 12-(9-anthroyloxy)-oleic acid; Di-4, Di-4 ANEPPDHQ; DIC, differential interference contrast; DRM, detergent-resistant membrane; EM, erythrocyte membrane; FLIM, fluorescence lifetime imaging microscopy; HBS, Hepes buffered saline; MβCD, methyl-β-cyclodextrin; pRBC, parasitized erythrocyte; nRBC, non-parasitized erythrocyte; nEM, non-parasitized EM; pEM, parasitized EM; PVM, parasitophorous vacuole membrane; PM, parasite membrane; TVN, tubulovesicular network.
